# The family meetings in oncology: some practical guidelines

**DOI:** 10.3389/fpsyg.2014.01552

**Published:** 2015-01-20

**Authors:** Paolo Gritti

**Affiliations:** Department of Mental Health, School of Medicine, Second University of Naples, Napoli, Italy

**Keywords:** psycho-oncology, family meetings, general systems theory, counseling, communication skills

## Abstract

Somatic illness is not only an individual experience of physical and psychological suffering, but also a psychosocial status that modulates the patient’s interpersonal relationships. Receiving a diagnosis of cancer causes severe distress. The patient’s family, too, feels the emotional ups and downs of the patient. Like the patient, they feel distressed during the onset, course and outcome of the disease. Minimizing the interpersonal impact of the illness contributes to an improved quality of life for both patients and caregivers. Thus, it is widely assumed that cancer treatments should include some kind of psychological support for the patient and family members. All of these treatments are aimed at improving collaboration and illness perception among family, patients and healthcare professionals, and support the family during the course of the disease and cancer therapies. The family system theory is a valuable framework to explain how the disease of the patient and the family’s daily life are interconnected. The therapeutic alliance with the family is a powerful tool to improve the quality of life for the patient, as well as to relieve the psychological distress of the family members who are involved. The following pages describe the objectives and conversational techniques that can be a tool for psychosocial work with the family of a cancer patient. The goal of this intervention is to help the patient’s family to understand their problems and acknowledge the anxiety and fear of mourning that can impede their capacity to face the everyday problems they must cope with. To achieve this goal, it is recommended that a meeting (or a series of meetings) be scheduled, and conducted both in hospital and in the home. The steps to set up and conduct a family meeting are described in the paper, with special emphasis on communication skills required to meet family expectations and discuss the crucial issues of their everyday life.

## INTRODUCTION

Somatic illness is not only an individual experience of physical and psychological suffering, but also a psychosocial status that modulates the patient’s interpersonal relationships. According to this conceptual framework, oncological diseases can be considered prototypes of the relational processes connected with the course and the outcome of all severe, weakening, chronic, or terminal illnesses (Shields et al., 2012). Receiving a diagnosis of cancer causes severe distress. The patient’s existential trajectory is entirely affected. Almost every cancer patient already knows the risks associated with the course of the disease, and of the treatments to be faced. However, the patient is never alone in the experience of illness. The patient’s family, too, experience the emotional ups and downs of the patient. Like the patient, they feel distressed during the onset, course and outcome of the disease (Baider et al., 2000). Minimizing the interpersonal impact of the illness contributes to an improved quality of life for both patients and caregivers (Northouse et al., 2013; Waldron et al., 2013). Therefore, it is widely accepted that cancer treatments should include some sort of psychological support for the patient and family members (Veach et al., 2002). Psychosocial oncology offers specific psychological models to help understand and address the interpersonal implications of neoplastic illnesses. Research findings suggest the potential benefit of psychological treatments for the partners and the relatives of the patient (Northouse et al., 2010). These interventions include a wide range of techniques and strategies placed into a theoretical continuum ranging from hermeneutic/interpretative to supportive/expressive, through to informative/educational models. All of these treatments are aimed at improving collaboration and illness perception among family, patients and healthcare professionals, and support the family during the course of the disease and cancer therapies. The focus is on specific issues such as the first diagnosis, treatment compliance, relapses, the oncologic child and, finally, the palliative care and the terminal phase. A main target of these interventions is the bereaved family (Kissane and Bloch, 2002).

## MEET THE CANCER PATIENT’S FAMILY: A SYSTEMIC-RELATIONAL APPROACH

The family system theory is a valuable theoretical framework to explain how the disease of the patient and the family’s daily life are interconnected (Mehta et al., 2009). According to the family systems approach, cancer can be considered to be an experience that involves the whole family; each family member is interdependent on the others to cope with the disease (Gritti, 2012). This paper targets the health professionals who spend at least part of their time supporting the cancer patient’s family as they struggle with the practical and emotional burden of their roles as co-carers. The therapeutic alliance with the family is a powerful tool to improve the quality of life for the patient, as well as to alleviate the psychological distress of the family members who are involved. The following pages describe the objectives and conversational techniques that can be a tool for psychosocial work with the family of a cancer patients (Moore et al., 2013; Bernacki and Block, 2014). These techniques are adapted and re-framed from the practice of systemic-relational counseling and psychotherapy. The goal of this intervention is to help the patient’s family to understand their problems and acknowledge the anxiety and fear of mourning that can hinder their capacity to face the everyday problems they must cope with. To achieve this goal it is recommended that a meeting (or a series of meetings) be scheduled, and conducted both in hospital and at the home (McDaniel et al., 1992; Griffith et al., 2004; Hudson et al., 2008; Powazki et al., 2014). In addition to the natural attitude and empathic traits present in the helping professions, such attributes must be enriched through training (Barth and Lannen, 2011), specifically through acquiring counseling skills.

## SET UP THE MEETING WITH THE FAMILY

### CO-ORDINATE THE SESSION WITH ONCOLOGISTS

It is clear that the appointment with the patient’s family, both in hospital and at home, can only be set up in advance with the oncologists. This is to prevent potential misunderstandings between doctors and family. Physicians are required to give information on the disease, the active treatment and the prognosis, and will be asked for explicit consent to the psychological meeting. This approach may seem overly cautious, but it protects against the risk of conflicting tensions between all parties involved in the clinical context. It seeks to avoid the possibility of negatively influencing the emotional disposition of the patient and the family—both of whom demand reassurance that they are part of a long-lasting and solid therapeutic alliance, in full harmony of purpose for the well-being of the patient.

### GATHER PRELIMINARY INFORMATION ON THE FAMILY

It is useful to acquire in advance some knowledge about the family, such as: composition, standard of living, residence, lifestyle, social life and, when possible, aspects of culture, values, and spiritual beliefs. This allows us to understand how the family will cope with the disease in terms of the material and psychological resources on which they will depend over the course of the disease, what expectations are placed on doctors and medicine, and also the resiliency or psychological vulnerability the family has demonstrated in the past.

### EVALUATING WHO TO INVITE TO THE MEETING

It is not always possible to have the participation of all family members at the meeting, depending on the cancer phases that may impose practical restrictions for the patient and relatives. You will need to evaluate at each stage, case who to invite, taking into account the fact that not everyone will be available. The patient, in particular, may be unable to participate because of health conditions. Even then, there is the opportunity to meet other family members. This could affect the topics of conversation in a completely different way. From time to time, it will be necessary to assess if this option might be preferable or, conversely, whether to postpone the meeting. In any case, take into account that scheduled meetings may not always go ahead as planned; flexibility is a prerequisite for those who choose to meet the patient’s family. The wisdom of including children during the meeting is open to question. On the one hand, they have their daily tasks, such as school and leisure time, which should be respected. On the other hand, they may present extreme distress when participating in the meeting, causing them psychological damage. However, in selected cases, the presence of children can be of great help relieve the anxiety of adults and to give a naturalness to the conversation. Of course, some issues will go unquestioned or may be focused on a version appropriate to the children’s understanding. When the patient is a child it is precisely the presence of a brother or sister which may become crucial. Naturally, the parents of child patients are prone to protect the siblings from the drama of the patient. Nevertheless, it must be borne in mind that children and adolescents are better able to support the experience of the patient’s illness if not everything is hidden from them. The hospital environment is not appropriate for a child; it is sometimes prohibited for them to attend the wards and surgeries, and the experience of the hospital may be even traumatic for them. So, it would be better to conduct the meeting at home.

### CHOOSE A PRIVATE SPACE FOR THE MEETING

It is a difficult challenge for the patients to describe to others their disease, to explain the ups and downs they encounter daily, and to express painful feelings. They would prefer to keep silence about this with their loved ones and, even more so, with strangers. Therefore, it is necessary to facilitate the conversation by providing a confidential ambience, and ensure the intimate nature of this experience. For example, choose a private place for the meeting, which is neither in a cancer care context or in a psychiatric environment. In some cases, the effectiveness of a meeting at home has been evident; at home, the family feels secure and even more available for the meeting. Furthermore, the home session gives the chance to see the everyday setting for the patient and to gather valuable information about family dynamics. These goals are easy to achieve in the patient’s home, whereas they are more difficult to attain in the hospital. Yet, with the consent of healthcare professionals, it is possible to get a similar effect during the hospital stay, by choosing a time of day in which it is easier to arrange for a quiet and comfortable place, where conversation will be easier. The space of the encounter should include easy chairs, sited in a circle to allow participants to face each other. The person leading the meeting should not have a privileged position, and each person should freely choose where to sit.

## THE STRUCTURE OF THE MEETING WITH THE FAMILY

The conversation with the family should adopt a very informal approach; a dialog inspired by a respectful listening and gentle guidance to explore the problems that the family is facing and enhance the opportunities for solutions (Gueguen et al., 2009; Hannon et al., 2012). However, it is appropriate to follow a fixed protocol for the meeting: the most relevant issues to raise, the communication skills to use, the attention to be given to the patient, the mistakes to avoid and, finally, the closing comments. It will be pleasant to begin with introductions and to clarify the premises and objectives of the meeting. A key aim of the meeting is to encourage the family’s active participation to treatments planning, and to check their appraisal of information provided by the oncologists. So, it will be very useful to investigate their actual knowledge about the patient’s illness. Because it is hard for the family to share an effective communication with care providers, this can give rise to misunderstanding. However, one must take care to avoid invading the oncologists’ domain. Patient and family should be encouraged to keep in regular contact with physicians about any unclear communication.

The middle phase of the conversation should explore the needs of the family. They are encouraged to share the questions still awaiting an answer from the health professionals. The counselor should ask the family for comments and opinions about ongoing treatments and therapeutic perspectives, reassuring them that he is committed to putting questions to the doctors and to giving the family clearer answers. This obligation to the family requires a good therapeutic alliance between the counselor and the oncologists, allowing the potential for researching the many expressions of disease experience of the family. At this point in the consultation, try to investigate carefully the emotions and beliefs that flow in the family, and try to back up the positive feelings of empathic pain sharing. Nevertheless, allow time to express negative emotions of anxiety and depressive pain. Finally, explore the problems that the family is forced to deal with in everyday life and the change in habits imposed by the illness. Offer the opportunity to discuss practical solutions to problems. The meeting should not neglect the most challenging issues about the experience of illness, though this should be included in a broader conversation about many other aspects of family life. The conversation should also focus on the present, observing the inevitable silence on the past and the anxiety about the future. Each family believes that many aspects of their lives cannot be shared with someone else, so it is necessary to respect the omissions in the narration of facts, circumstances and emotional ties. Try to give up the language of medicine, psychiatry and psychology, and to attune with the everyday language of the family. Use clear and open questions but never direct or intrusive ones (Dumont and Kissane, 2009). Be prudent when commenting, and make it clear that these are a preliminary point of view. Be prepared to repeat or re-phrase questions and comments, as it is useful when dealing with emotions and experiences that are hard to understand. Respect brief silences, should they occur; do not interrupt the speaker; allow people to express themselves, but without feeling under pressure to talk; do not underestimate the aspects of non-verbal communication. Remember that the meeting with the family is oriented to ease the expression of emotions and feelings, and investigates the resources the family can rely on difficult circumstances. As a result, the conversation will encourage the family to express memories, metaphors and images, and subjective meanings of the disease. Keep a listening attitude on these issues, avoiding any interpretation as it does not fall under the scope of this single encounter with the family. Support the patient, who remains at the center of the conversation, and emphasize his/her own suffering, communication difficulties, commitment to active participation in treatment and, finally, the influence of his/her emotional swings on relatives. Experience of counseling has shown that the patient constantly shifts from the expectation of an endless support by the family members, to the effort to be autonomous in the management of the disease. The following paragraphs describe in detail each of the points mentioned above.

## MEETING THEMES

### SHARE INFORMATION ABOUT THE DISEASE

In the initial phase of the meeting it is always appropriate to ask the family what their understanding of the disease is, the sources of such knowledge and whether they need to obtain more information. However, the therapist should be aware that each member of the family has partial information that may differ from others—and that only the key caregiver knows all the relevant information. This issue should be explored with caution, considering the implicit need of some family members, to keep confidentiality about certain information.

### INQUIRE HOW THE FAMILY IS COPING WITH THE DISEASE

An important point of the meeting will be focused on the description of the coping strategies required to face the illness. It will be pertinent to investigate both solutions shared by all, as well as the personal solutions of each of them. They will be questioned about the effectiveness of the daily processes of adaptation and helped to develop new solutions to the current problems. The therapist should support the family in the search for pragmatic solutions and emotional energies within the network of daily relationships. This will buffer their feelings of isolation and abandonment with optimistic expressions about people’s willingness to intervene in support of their ongoing problems.

### PROVIDE TIPS OR ADVICE ON BEHAVIORS OR CHOICES

Frequently, the family is struggling with having to reach hard decisions or coping with unexpected or complex issues of life. In this case, the family will welcome tips and advice, as well as exchanging ideas with the therapist, who can examine all aspects of their decision-making dilemmas. In this case, the best strategy is to argue with them all the positive and negative aspects of the alternatives that they face to help them reach a decision, mindful of the risks and opportunities that every choice entails.

### DISCUSS THE QUALITY OF CARE

It will be essential to consult the family about their views on cancer treatments. This topic will be dealt with only in the context of helping the family and the patient to strengthen the therapeutic alliance with the healthcare system. Inappropriate expectations about the treatment outcome, the adverse effects of the treatments, the choice and timing of each treatment may be expressed by the family in such a way as equips the therapist to assume responsibility for an effective communication with health professionals.

### DISCUSS THE QUALITY OF THE RELATIONSHIP WITH THE DOCTORS

It is good to give the family an opportunity to express opinions on the quality of relationships with health professionals. The meeting should provide a neutral position, offering a safe environment where they will be able to express openly the positive or negative aspects of the doctor–patient–family relationship.

### INVESTIGATE THE FEELINGS OF FAMILY MEMBERS TOWARD THE PATIENT

The central part of the meeting should be dedicated to facilitating the expression of relatives’ moods, emotions and feelings associated with the experience of illness. It should be kept in mind that the patient’s physical and psychological suffering generates complex and contradictory feelings within the family. On one hand, a full sharing and some of the patient’s suffering, will be observed; on the other hand, there is an almost emotional detachment—an indifference—in response to trying to remove from consciousness an untenable anxiety about the present and the future of the patient. Anger against the disease, interpreted as the result of a tragedy or of a superior will toward the patient, whose sufferings are always heavy to sustain on the practical, affective and spiritual side; and, finally, toward medicine and doctors, considered inadequate to provide a complete, final hope of recovery.

### EXPLORE THE PATIENT’S FEELINGS TOWARD FAMILY MEMBERS

Similarly, the adult patient will be plagued by conflicting feelings toward his family, swinging from the expectation of a constant presence and material support to the desire to relieve their commitment and their obvious distress. The outcome of these psychological dynamics, sometimes, will induce an observable state of depression, discouragement and pessimism or, on the contrary, an apparent serenity, and even the ability to give emotional support to relatives.

### GIVING A NAME TO EACH PERSON’S EMOTIONS

It will be of great psychological help to give a name to the confused feelings expressed by the family. For this reason, the therapist should be a person who, from time to time is given to describing him/herself using his own feelings with the most relevant and appropriate expression. The effect of this exercise will benefit the subjective awareness of each family member. For example, since the word anxiety is extremely frequent during the conversation, it will be very useful to translate this broad and ambiguous word to the most appropriate expression to describe the specific emotional experience for everyone.

## SOME CONVERSATIONAL STRATEGIES

### DO NOT AVOID DISCUSSING THE HARD ISSUES ABOUT THE DISEASE

Families’ daily conversations often elude all topics related to the disease, to the treatment and to the outlook of care. This shared decision is quite understandable if one takes into consideration the needs of the family to maintain some form of normal life, whereas the disease is experienced as a traumatic and destructive event. However, during the meeting, it will be helpful to try to urge the family to reflect further upon the experience of cancer and its consequences in the lives of everyone. The silence on these issues, in fact, increases the likelihood of pernicious ghosts of pain and death.

### DO NOT LIMIT YOURSELF TO THE CHALLENGING ISSUES OF THE DISEASE

It will be fruitful to discuss many other aspects of family life. To dwell on trivial topics, to smile about daily and grotesque events, to ask about their habits and interests, will help to scatter the distressing fog that the disease generates in family life.

### INVITE TO SPEAK BUT NOT FORCE TO SPEAK

The conversation with the family should be oriented by an empathic emotional tone and focused on expressing what is not easy to say. Nevertheless, this solicitation will still have to take into account the inhibition of the family to express aspects of their lives they consider to be very private, as well as their natural disinclination to “wash their dirty linen” in the family. So the therapist should be further able to discern if and when persisting on topics causes an excessive embarrassment or discomfort. For example, the issue of physical intimacy within the couple, which is likewise affected by certain neoplastic diseases.

### RESPECT PRIVACY AND SECRETS

The therapist’s personal sensitivity will guide him in respect of those events of family life, not necessarily linked to the cancer, which are part of the family’s shared secrets. Family secrets are normative features of family life: painful or simply embarrassing facts, circumstances concerning family, ancestors or relatives removed from the conscience. Since the meeting with the family originates from the experience of illness, and is focused on it, these aspects of family biography should be omitted.

### FOCUS ON THE PROBLEM

The conversation with the family will be predominantly oriented to examine the contingencies of the current family life, particularly, those connected with the disease. Disease guides the psychological processes of all who are involved in it. Some patients and their families are reassured by the daily duties because it fuels the illusory belief that the time has stopped, as frozen in the present and, simultaneously, the course of the disease.

### RESPECT THE SILENCE ABOUT THE PAST AND ANXIETY ABOUT THE FUTURE

These considerations give an account of the opportunity to avoid forcing the family to recall the past. This applies not only to the painful or unpleasant memories, but also to the progressive and happy events of the past. All the past, when compared with the present life, could be burdensome to the patient and family. The future of cancer patients is never a source of optimism. The word “cancer” is always, though erroneously, connected with the word “death.” So the future of the family needs to be measured as the probability, still so frequent, that the disease will prevail on the care, and that life expectancy is short—or much shorter than one would want. As a result, any discussion about the future will be burdened by this agonizing wait to find out if, and when, the patient will recover; what will happen to the family in either case; and how to face the terrible and disastrous event of the patient’s death. The meeting with the family should concern all these issues only if the family shows a degree of psychological resilience to these problems.

## COMMUNICATION SKILLS

The meeting requires communication skills specific to the premise and the context, described below.

### GIVING EMOTIONAL SUPPORT AND ALLEVIATING DISTRESSING OR MOURNFUL FEELINGS

The therapist should be able to give hope and optimism about the outcome of the treatments and the prognosis of the disease. To achieve this purpose a resilient personality, forged by the vicissitudes of his or her existence, is required as well as a positive expectation on the emotional resources of people.

### PROVIDING INFORMATION

If prompted, you should try to explore the clinical record acquired by the patient and family, ensuring they do not differ from those provided by health professionals. Therefore, it is necessary to collect the essential information directly from the oncologists regarding the diagnosis, treatment and prognosis. Should the oncologists be reluctant to supply this information (citing the privacy rights of the patient), then it would be best to suggest the family members to refer to them only.

### HELPING THE FAMILY TO RESOLVE CONCRETE PROBLEMS

It can be very useful to the patient and family to receive help in dealing with current problems of daily life. It is also helpful to discuss possible solutions and the concomitant difficulties with a neutral participant whose opinions are guided by common sense. The therapist should be careful to share with the family all the feasible alternatives in order to help them make a choice.

### GIVING SUGGESTIONS OR RECOMMENDATIONS

The family therapist, in the position of the “Good Samaritan,” should provide hints and recommendations that accept that the actual everyday life of the family may not coincide with his/her own experience of the effects of neoplastic disease on people’s lives.

### PROMOTING CONTACT WITH THE REALITY OF THE DISEASE

One important function of emotional support for the family is the ability to guide people to a progressive and full awareness of the disease. In doing so, bear in mind that a natural defensive response of the patient and family includes the negation of the events associated with the onset of symptoms, the diagnostic phase and the effects of cancer treatment.

## COMMUNICATING CLEARLY

### CHOOSE A SIMPLE AND EFFECTIVE LANGUAGE

The easiest and most effective way of building a constructive conversation with the patient’s family is the use of their everyday language. Sometimes, oncologists, psychiatrists and psychologists are forced to use words oncerning their professional skills when formulating a diagnosis or implementing a treatment. In contrast, the therapist should be free from these obligations and should use language appropriate to the family setting. This helps to establish a confidential climate during the meeting. In some cases, it may even be helpful to use the colloquial language preferred by some families. It should be possible to formulate direct questions regarding topics of the conversation—however, these should not be too intrusive. Questions should focus on the topic, summarize the content of the argument, and be sensitive to the opportunity for frankness. In general, direct questions about issues related to anxieties about the disease should be avoided; however, those concerning the increased economic burden as a result of the disease should be addressed directly.

### ASKING “OPEN” QUESTIONS

Use questions that allow more than a “yes” or “no” response (“open”) questions. This question style can open a wider, articulated dialog, leaving room for personal views.

### COMMENTING IN A USEFUL AND CONSTRUCTIVE WAY

Brief, clear and concise comments, tips and suggestions are worthwhile. Often the patient and the relatives are waiting for others’ perspectives on the day-to-day choices to rely on. The comments of the interlocutor should be built on trust and optimism, even at the cost of some innocent lie on the actual severity of the disease and its prognosis. Instill hope, energy, promotes more adaptive resources to better face the disease, remains the main aim of meeting the patient and his family.

### REPEAT AND RE-PHRASE

Cancer is a profound psychological trauma for both the patient and his family members. This traumatic experience is renewed during each of the fundamental stages in the course of the disease. As the psychological impact of the trauma affects cognitive function, the decision-making processes, to decide what can be done to cope with the disease, are lacking. Therefore, communication between healthcare professionals, patient and family may be ineffective. To obviate this risk, it is prudent to assume that our conversational partners cannot always understand fully what is being said to them. So be prepared to repeat information and concepts, to ensure that the patient and family understand the meaning of what is being said. Any questions and statements should be rephrased in a simpler way. Finally, at the end of the meeting, it is useful to summarize what has been discussed.

### USE ACTIVE LISTENING

Active listening and empathy are the basic dowry of the therapist who meets the family. Initially, he/she should be able to accept what the patient and family want to express without interrupting or rushing to comment on everything that is said. The therapist must prepare allow the inevitable moments of silence on the most troublesome issues, but must also able to break a long silence and turn the conversation to a less distressing topic. Active listening allows the therapist to grasp the emotions underlying the dialog. Images and metaphors that may arise during the conversation—both used to describe deep content that cannot be otherwise expressed—are also best served by active listening. Finally, active listening will also help in understanding the existential and spiritual experience that the disease elicits in the family.

### BE ALERT TO NON-VERBAL COMMUNICATION

Finally, the observation of non-verbal communication may be useful to improve the understanding of the aspects described so far. The mimicry of the face (often more candid than words), the posture, the looks, the gestures, the use of space, even the style of clothing can guide the therapist in gaining knowledge of the family.

### AVOID MISTAKES

The patient’s family participating in the meeting are eager to listen and find shelter for their problems; they expect to meet a person driven not only by the desire to provide a practical, psychological, as well as spiritual, aid but also a competent person, guided by his/her own experience and by subject-specific training. However, these expectations, although positive, can be undermined by a number of mistakes in the management of the meeting. Among the most frequent errors in meeting management are:

• completely ignoring a meeting participant
• being too demanding regarding the meeting objectives
• disqualifying or negatively connoting opinions or sentiments expressed
• giving priority to dialog to specific family members
• feigning emotional involvement
• implying resignation
• evading questions
• expressing a psychological/psychiatric diagnosis.

## THE SERIES OF MEETINGS WITH THE FAMILY

When, after this initial meeting, the family is available for more appointments, the therapist’s work should include more specific psychological skills focused on counseling. A counseling process with the patient’s family should pursue the following objectives:

(a) strengthening the emotional ties to soothe the anguish of the patient and the family faced with the outlook of physical suffering, and problems of impending, although transient, separation; finding new emotional resources for all members
(b) reassuring the patient, who fears an affective detachment from the family or from their partner, resulting in the fear of deprivation of bodily intimacy or even a tacit physical or emotional betrayal
(c) sustaining family members in the commitment to daily support emotional and practical needs of the patient, assisting them in reassigning physical and psychic energy to the patient, without neglecting other affective or working duties
(d) supporting the family in maintaining constant emotional ties with kinship and friends, thus avoiding social isolation.

Scheduling a meeting or series of meetings with the family at least twice during the disease is recommended. First, during the phase of emotive shock that follows the communication of diagnosis and the treatment planning. This action is called “counseling on emotional crisis.” It is possible that only two or three meetings can help the family build up effective strategies to face the disease and its consequences for family life. It may also support the family in building a therapeutic alliance with the doctors, facilitating the communication process between them. In this sense, the therapist acts as a “mediator of the relationship” between the two parties. The module called “brief counseling” can be proposed during the advanced phases of the cancer. It consists of two to four meetings aimed at supporting the coping processes, as well as facilitating emotional expressiveness, including those of anger and guilt. Finally, the “bereavement counseling” module is targeted at the emotional comfort of family members during the mourning after the death of the patient, and also to help the family to move forward. If necessary, these meetings can be repeated.

## CONCLUSION

In our experience the aforementioned communication skills and conversational techniques guiding meetings with cancer patient’s families are a useful tool to address their distress if routinely included in psychosocial support programs in oncology. A pilot study is in progress in order to assess the effectiveness of this format of family meetings. A cohort of cancer patients, referred for psychiatric consultation before undergoing chemotherapy, are screened with the Italian validated version of the Distress Thermometer (Grassi et al., 2013). The key family caregiver distress is screened as well. Patients and their caregivers affected by severe distress (cutoff score ≥5) are recruited for the study. The study arm is assigned to a family support program consisting of four weekly family meetings whereas a four-session psychiatric consultation is given, as usual, to the control arm. A retest is conducted at the end of the intervention and a month later. Preliminary observational data, conducted on 10 cases, suggest a moderate buffering effect of family meetings on the patient as well as on the family caregiver distress if compared with the benefit of psychiatric consultation.

### Conflict of Interest Statement

The author declares that the research was conducted in the absence of any commercial or financial relationships that could be construed as a potential conflict of interest.
